# Respiratory Health and Related Quality of Life in Patients with Congenital Agammaglobulinemia in the Northern Region of the UK

**DOI:** 10.1007/s10875-016-0284-3

**Published:** 2016-04-18

**Authors:** Branwen A. Bryan, Alex Battersby, Benjamin Martin James Shillitoe, Dawn Barge, Helen Bourne, Terry Flood, Andrew J. Cant, Catherine Stroud, Andrew R. Gennery

**Affiliations:** Institute of Cellular Medicine, Newcastle University, 4th Floor, William Leech Building, Medical School, Framlington Place, Newcastle upon Tyne, E2 4HH UK; Paediatric Immunology, Great North Children’s Hospital, Queen Victoria Road, Newcastle upon Tyne, NE1 4LP UK; Great North Children’s Hospital, Clinical Resource Building, Level 4, Block 2, Newcastle upon Tyne, NE1 4LP UK

**Keywords:** XLA, agammaglobulinemia, immunoglobulin, bronchiectasis, quality of life

## Abstract

**Introduction:**

Patients with congenital agammaglobulinemia, characterized by a defect in B lymphocyte differentiation causing B alymphocytosis, require life-long IgG replacement. There is scant literature regarding the effectiveness of IgG treatment at preventing mucosal (particularly sinopulmonary tract) infection and whether current management adequately restores “normal” health and quality of life (QoL). We aimed to document infective episodes pre- and post-commencing IgG replacement, determine any change in lung function and structure and assess respiratory status and QoL in a cohort of patients treated in Newcastle.

**Methods:**

Clinical data were extracted from medical records of 15 patients identified from the immunology database, focusing on infective episodes, serial chest CT and spirometry results. Thirteen patients completed a selection of standardized and validated questionnaires assessing physical health, respiratory health and QoL.

**Results:**

Pediatric patients on IgG therapy suffered fewer infections per patient year (0.74) than adults (2.13). 6/14 patients showed deteriorating respiratory status despite adequate therapy. Health questionnaires revealed a significant burden of respiratory disease on a patient’s life.

**Conclusion:**

Clinical data showed patients with congenital agammaglobulinemia receiving immunoglobulin therapy retained a higher than average infection rate, most of which affected mucosal barriers. Most patients self-reported worse respiratory symptoms, a lower respiratory-related QoL and a lower general health QoL relative to a healthy population. Most participants had progressive structural lung damage and decreased lung function. These results suggest that current management is not entirely effective at preventing deterioration of respiratory health or restoring QoL.

## Introduction

X-linked agammaglobulinemia (XLA) accounts for 85 % of cases of primary agammaglobulinemia [1–3]. It is due to mutations in Bruton’s tyrosine kinase (BTK) which encodes a hematopoietic-specific enzyme playing a critical role in B lymphocyte development and receptor signaling [1–3]. Other patients can suffer from recognized autosomal recessive forms (e.g. μ chain) [4]. The resulting agammaglobulinemia significantly contributes to the clinical characteristics, as immunoglobulins are pivotal in the humoral response, particularly in protecting against encapsulated bacteria [5, 6]. Patients are adequately protected against most non-bacterial pathogens, with the exception of enteroviruses, which can cause severe meningoencephalitis, arthritis or enteritis, and *Giardia lamblia*, which causes enteritis [7–9]. The mean age at diagnosis of XLA has improved overtime to 3.5 years old, with a clear correlation between prompt diagnosis and improved long-term patient outcome due to reduced baseline end-organ damage from early infections [3, 7, 10].

Treatment involves life-long replacement of IgG, either subcutaneously or intravenous, and judicious prophylactic antibiotic use. Patients on immunoglobulin replacement therapy have reduced episodes of systemic infection such as encephalitis, meningoencephalitis and sepsis [7]. However, the efficacy in reducing mucosal infections, in particular those affecting the sinopulmonary tract, is less clear. Some studies have suggested that repeated low-grade infections of the sinopulmonary tract can lead to significant, progressive chronic lung disease and hearing difficulties [11, 12]. When first described, life expectancy for patients with XLA was in the early 20s but now patients survive into their fourth decade and beyond [13, 14]. However, there are few data on the long-term impact of this condition and its management on patients’ outcomes, for example, frequency of mucosal infections, progressive lung damage and health-related quality of life (HRQoL).

This retrospective, cross-sectional study of patients with XLA and autosomal recessive agammaglobulinemia in the northern region of England documented the number and type of infective episodes both pre- and post-instigation of immunoglobulin replacement therapy, and assessed respiratory status and health, with lung function alongside radiological investigations, and determined evidence of progressive respiratory deterioration. The subsequent impact on quality of life of patients and their careers was also assessed.

## Methods

Patients in the northern region of England who had a definitive or probable diagnosis of XLA as per the PAGID guidelines were included [15].

Definitive: less than 2 % CD19^*+*^ B lymphocytes and at least one of the following:

- Mutation in Btk
- Absent or diminished Btk protein expression
- Maternal cousins, uncles or nephews with less than 2 % CD19^+^ B lymphocytes

Probable: less than 2 % CD19^*+*^ B lymphocytes and at least one of the following:

- Onset of recurrent bacterial infections in the first 5 years of life
- Serum IgG, IgM and IgA more than 2 SD normal for age
- Absent isohemagglutinins and/or poor response to vaccines
- Other causes of hypogammaglobulinemia have been excluded

In addition, patients with proven autosomal recessive agammaglobulinemias (e.g. μ heavy chain deficiency) and less than 2 % CD19^+^ lymphocytes were included.

Assessment of current management of patients was conducted using a standard proforma to collect information from medical records. Presentation and age at diagnosis, infective signs and symptoms pre- and post-diagnosis, and initiation of Ig therapy, other treatment received, hospital admissions and any surgical treatment were recorded. Where available, laboratory analysis of current B lymphocyte count, IgG trough levels and BTK protein expression were also recorded.

Age of diagnosis was recorded according to clinical judgment and not molecular analysis, as clinical judgment coincided with Ig therapy instigation, whereas molecular diagnosis was often confirmed much later.

An infective episode was recorded if symptoms were suggestive of an infection and antibiotics were prescribed. For example, an episode with productive cough, colored sputum, temperature and general malaise would be recorded as a respiratory tract infection. If no symptoms were documented but the diagnosis was recorded as an infection and treatment was antibiotics, this was also recorded as an infection. When notes recorded “recurrent” infections, an arbitrary frequency of three infections per year was assigned. Due to the lack of detail provided, this enabled a representation of the minimum frequency of infections implied while maintaining consistency throughout data collection.

Patients’ clinical information was gathered and compared per individual pre- and post-Ig replacement therapy. Particular attention was paid to any relevant investigations, inter alia, high-resolution thoracic computerized tomography (HRCT), chest radiographs and pulmonary function tests. Sequential thoracic computerized tomography imaging reports were compared from the initial scan to the most current, in order to assess any level of change and any baseline damage. A similar approach was taken when assessing pulmonary function results; FEV1 and FVC values were compared to the predicted value, and this difference was then used to monitor change over time. If the gap between the actual lung function and the predicted value increased, this was used to indicate deterioration. If the lung function was below the normal predicted value but no change occurred, this was recorded as stable abnormal. If the difference between the achieved lung function and the predicted value decreased, this was used to show improved abnormal. If the lung function achieved represented the predicted value, this was reported as normal lung function. Normal reference ranges, inter-test variability and changes due to age were taken into account in this analysis [16, 17].

The St George’s Respiratory Questionnaire (SGRQ) was used to examine respiratory health. This is a well-recognized tool, originally designed for patients with COPD and asthma, but since used and validated in a number of studies for patients with bronchiectasis [18–20] and has been used in patients with primary antibody deficiency [21]. The SGRQ assesses three components of respiratory health: symptoms, activity and impact. These scores were combined and the total compared to a healthy person, equivalent to a score of 0. Therefore, the higher the score that was achieved, the worse the health impairment was. This data was compared against population norms [22].

The Short-Form 36 version 2 (SF-36v2) [23] was used to assess quality of life for patients aged 16 years and older. This is a widely used tool to measure quality of life, is well validated and has been used in primary immunodeficiency [13, 24]. The SF-36v2 comprises of eight components, each scored out of 100, with the best score being 100. These were combined to give a physical health score and a mental health score. These data were compared against UK published norms [25].

Ethical approval was gained from the NRES committee North East, Newcastle and North Tyneside 2, and written informed consent was received from all participants prior to enrolment.

### Statistical Analysis

Data were analyzed in spss.v22 (www.spss.com). Non-parametric data were displayed as median and range and tested against population norms using the one-sample Wilcoxon signed-rank test. Parametric data were displayed as mean and standard deviation and differences were tested against population groups using one-sample *t* test. Differences between groups within the cohort were tested using the Mann-Whitney *U* test.

## Results

### Patients

Patients with congenital agammaglobulinemia were diagnosed and managed by the regional immunology center at the Royal Victoria Infirmary (adults) or the Great North Children’s Hospital, Newcastle upon Tyne. Fifteen patients were identified from the local database, of which 14 had XLA and 1 had mu chain-deficient autosomal recessive agammaglobulinemia. Available medical records of the 15 patients were analyzed providing approximately 150 cumulative years of past medical history.

The median age of study subjects was 26 years (range 5–46); 9 were adults (range 22–46 years) and 6 children (range 5–16 years).

The median age at diagnosis in pediatric patients was 33 months (range 16–49 months). Age at diagnosis was available for 5 of the 9 adult patients with a median age at diagnosis of 64 months (range 57–110 months). Therefore, the overall median age at diagnosis was 49 months (range 16–110 months). The median follow-up for pediatric patients was 8 years 6 months, for adults (for whom data was available) 18 years 2 months with and an overall median follow-up for the cohort of 10 years 6 months.

Three families had more than one affected child included in the study, meaning that for 3 patients, there was a positive family history (patients 10 and 12, 8 and 15, 11 and 13). However, in none of the families was the second child screened and all boys were subsequently symptomatic at time of diagnosis. Therefore, all patients were diagnosed on clinical history and symptoms.

Four patients had a mutation identified in *BTK*, a further 3 had absent BTK expression but no mutation was recorded. Gene mutation data was unavailable for 6 patients. The one female in our cohort had absent mu chain expression. There were two patients with apparent normal BTK expression and no mutation identified. However, these two patients were brothers and diagnosed on this basis along with absent B lymphocytes. All except one (with very low B lymphocyte numbers—patient 9) had absent B lymphocytes and all those for whom data at diagnosis was available (6 patients) had low or absent immunoglobulins (Table 1).

**Table 1:** Patient characteristics, Ig levels at diagnosis, B lymphocyte percentages, BTK expression and presence of mutation

Patient number	Age	Immunoglobulin levels at diagnosis (g/dl)			CD19^+^ B lymphocytes^a^		BTK expression	Mutation
		IgG	IgA	IgM	Diagnosis	Current		
1	Pediatric	0.34	<0.05	0.084	<1 %	<1 %	Absent	N/A
2	Pediatric	<1.5	<0.02	<0.3	<1 %	<1 %	Diminished	Unknown
3	Pediatric	N/A	N/A	N/A	N/A	<1 %	Absent	Unknown
4	Pediatric	<0.33	<0.07	<0.11	<1 %	<1 %	Absent	Yes (c.877C>T)
5	Pediatric	<0.33	<0.7	<0.08	<2 %	<1 %	Absent	N/A
6	Pediatric	0.5	<0.07	0.33	<1 %	<1 %	Absent	Yes (c.1911delA)
7	Adult	N/A	N/A	N/A	<1 %	<1 %	Absent	Yes (R225X)
8	Adult	N/A	N/A	N/A	N/A	<1 %	Absent	Unknown
9	Adult	N/A	N/A	N/A	N/A	6 %	Absent	No
10	Adult	N/A	N/A	N/A	N/A	<1 %	Normal	N/A
11	Adult	N/A	N/A	N/A	N/A	<1 %	Absent	Unknown
12	Adult	N/A	N/A	N/A	<1 %	<1 %	Normal	N/A
13	Adult	N/A	N/A	N/A	7 %	<1 %	Absent	Unknown
14	Adult	N/A	N/A	N/A	N/A	<1 %	Absent	Yes (c.863G>A)
15	Adult	2.9	<0.48	0.15	N/A	<1 %	N/A	Unknown

*N*/*A* not available

^a^As percentage of total lymphocyte count

### Prophylactic Therapies

Seven (3 children, 4 adults) out of the 15 patients received intravenous immunoglobulin therapy (IVIG) with the remaining 8 (3 children, 5 adults) on subcutaneous immunoglobulin (SCIG). Fourteen out of 15 patients had persistent IgG trough levels >6 g/dl (5 of whom had trough levels of 6–7 g/dl, 9 of whom had trough levels >8 g/dl). There was one adult patient with recent persistent IgG trough levels of 4–5 g/dl; this patient showed slight progress of minor bronchiectasis on HRCT scans. There was no significant difference in IgG trough levels between those on SC and those on IV replacement therapy (*p* = 0.385).

### Infections

All patients continued to experience infections after commencing immunoglobulin therapy, the majority of which were mucosal and affected the sinopulmonary tract. However, no patient experienced invasive or systemic infections, while there were seven such episodes prior to starting immunoglobulin therapy (Table 2).

**Table 2:** Total number and type of infections pre- and post-instigation of replacement immunoglobulin therapy (mean time to diagnosis = 49 months, mean follow-up time = 14.7 years)

Infection type	Number of infections	
	Pre-immunoglobulin therapy	Post-immunoglobulin therapy
Sinopulmonary		
Chest	28	61
Sinus	0	30
Ear	8	5
Skin	15	13
Eyes	5	7
GI	3	12
CNS	4	0
Sepsis	2	0
TB	1	0
Bone/joint	2	0

The pediatric infection rate on immunoglobulin therapy dropped from 2.9 to 0.74 infections/patient/year. (The dataset was incomplete to determine the number of infections/year pre-immunoglobulin administration for the adult patients.) The pediatric infection rate while receiving immunoglobulin therapy was lower than that for adults receiving immunoglobulin therapy (0.74 vs 2.12 infections/patient/year).

The proportion of infections that were mucosal increased following commencement of immunoglobulin replacement in both pediatric (36 to 66 %) and adult (59 to 81 %) patients. Specifically, the proportion of sinopulmonary infections as a percentage of total infections increased post-immunoglobulin therapy in pediatric (36 to 50 %) and adult (43 to 51 %) patients.

### Clinical Imaging and Lung Function

Successive computerized tomography thoracic images were available for 14 participants. Eight patients had evidence of bronchiectasis, 5 of whom showed progression of their lung disease while on immunoglobulin replacement therapy. There was pulmonary function data available for 12 patients, 2 of whom had deteriorating pulmonary function tests while on immunoglobulin therapy (Table 3). Of the 14 patients for whom radiological and/or respiratory function information was available, 8 had radiological evidence of bronchiectasis of which 6 demonstrated either functional and/or structural pulmonary deterioration after diagnosis and commencement of immunoglobulin therapy. One patient in our cohort suffered from severe bronchiectasis as result of his XLA and underwent a lung transplantation. Out of the 6 patients who showed a worsening of their bronchiectasis, 4 had IgG trough levels consistently >10 g/dl (Table 4). IgG trough levels tended to be higher in those patients with bronchiectasis but this did not reach statistical difference (*p* = 0.073).

**Table 3:** Patient’s lung function tests

Patient number	Current lung function^a^			Previous lung function^b^		
	FEV1 (% predicted)	FVC (% predicted)	FEV1/FVC ratio	FEV1 (% predicted)	FVC (% predicted)	FEV1/FVC ratio
1	N/A	N/A	N/A	N/A	N/A	N/A
2	N/A	N/A	N/A	N/A	N/A	N/A
3	1.70 (72 %)	2.05 (78 %)	82 %	1.5 (69 %)	2.1 (86 %)	71 %
4	1.94 (72 %)	2.42 (79 %)	77 %	0.85 (59 %)	1.08 (70 %)	63 %
5	2.1 (50 %)	3.25 (66 %)	59 %	1.1 (51 %)	1.6 (64 %)	63 %
6	2.0 (90 %)	2.4 (92 %)	83 %	1.55 (83 %)	1.65 (76 %)	94 %
7	4.1 (85 %)	4.15 (73 %)	99 %	3.28 (79 %)	3.62 (75 %)	91 %
8	3.37 (84 %)	N/A	N/A	3.54 (88 %)	3.96 (85 %)	89 %
9	2.75 (88 %)	4.79 (95 %)	78 %	3.85 (90 %)	5.0 (100 %)	77 %
10	4.2 (98 %)	5.35 (105 %)	79 %	3.7 (86 %)	5.01 (98 %)	74 %
11	3.43 (93 %)	4.02 (113 %)	85 %	3.46 (93 %)	4.65 (106 %)	74 %
12	3.2	4.13	77 %	N/A	N/A	N/A
13^c^	0.75 (21 %)	1.53 (37 %)	49 %	0.82 (23 %)	1.55 (37 %)	53 %
14	3.5 (90 %)	4.0 (93 %)	79 %	3.42 (83 %)	4.3 (85 %)	80 %
15	N/A	N/A	N/A	N/A	N/A	N/A

*N*/*A* not available

^a^Latest lung function from 2010 to 2012

^b^Previous lung function from 2005 to 2007

^c^Patient 13’s lung function pre-transplant

**Table 4:** Ig route, trough levels, lung disease and co-morbidities for each patient

Patient number	Ig route	IgG trough level (g/dl)^a^	Lung disease^b^		Prophylactic antibiotics	Co-morbidities
			CT	Lung function		
1	IV	7.4	Not done	Not done	None	Gastro-esophageal reflux
2	SC	7.1	Normal	Not done	None	Eczema
3	SC	8.7	Progression	Stable—obstructive	None	
4	IV	11.7	Stable disease	Stable—obstructive	Azithromycin 3×/week	
5	SC	7.6	Stable disease	Stable—obstructive	None	
6	SC	7.9	Normal	Stable—normal	None	
7	SC	5.8	Progression	Stable—restrictive	None	
8	SC	1.6	Stable disease	Deterioration—obstructive	None	
9	IV	6.9	Normal	Stable—normal	None	Deafness
10	SC	9.0	Normal	Stable—normal	None	
11	IV	7.6	Normal	Stable—normal	None	
12	SC	9.5	Normal	Stable—normal	Azithromycin 3×/week	
13	IV	11.5	Progression (pre-transplant)	Deterioration—obstructive (pre-transplant)	Co-trimoxazole daily	Lung transplant
14	IV	10.0	Progression	Stable—normal	None	
15	IV	15.3	Progression	Not done	None	Autism, epilepsy

^a^Mean over the last 5 years

^b^From the previous 5 years

### St George’s Respiratory Questionnaire

Questionnaire data were collected from 12 patients; 1 was unavailable to contact, 1 failed to return the questionnaires and 1 patient was incapable due to significant learning difficulties secondary to meningitis. SGRQ scores were compared against published normative data for Spanish males aged 40–49 (Table 5) [22].

**Table 5:** Breakdown of SGRQ compared against normative data (completed by 12 patients) and further broken down to compare those with and without bronchiectasis. Scores are displayed as median (range)

Component	Score (all patients)	Population norm	*p* value (all vs norm)	Score (with bronchiectasis)	*p* values (vs norm)	Score (no disease)	*p* values (vs norm)	*p* values (disease vs none)
Symptoms	33.8 (6.32–83.59)	4.24 (0.0–32.60)	0.020	43.98 (13.34–83.59)	0.028	25.08 (6.32–62.75)	0.028	0.522
Activity	20.83 (0.0–92.51)	0.0 (0.0–29.31)	0.012	36.43 (0.0–92.51)	0.043	5.61 (0.0–31.42)	0.109	0.042
Impact	8.83 (0.0–50.64)	0.0 (0–15.1)	0.003	15.63 (6.68–50.64)	0.028	4.74 (0.0–21.18)	0.043	0.055
Total	17.21 (1.05–64.65)	2.72 (0–22.62)	0.005	27.78 (10.64–64.65)	0.028	8.38 (1.05–31.18)	0.116	0.055

The SGRQ showed a median score of 17.21 (1.05–64.65), significantly higher than the published population normative median of 2.72 (0–22.62, *p* = 0.005). Each component of the SGRQ was significantly higher than population norms (Table 5), indicating a higher rate of respiratory disease and burden than a healthy population. Patients with bronchiectasis had significantly worse SGRQ scores in all domains compared to a healthy population. Those without bronchiectasis had significantly worse scores than a healthy population only in symptom and impact domains. Compared to the patients without bronchiectasis, those with bronchiectasis had significantly worse scores for activity and tended to have to a worse activity and overall total score (*p* = 0.055 in both domains). There was no significant difference in the symptom scores between the two groups (*p* = 0.522).

### Short-Form 36 Version 2

SF-36v2 questionnaires were returned for all 9 adult participants; however, one form was incomplete and therefore excluded from further analyses. The results showed no significant difference between the patients’ QoL compared to healthy UK norms, except for the component ‘General Health’ which was significantly worse in the congenital agammaglobulinemia group (59.5 (SD 30.25) vs 78.3 (SD 15.61), *p* = 0.012) [25] (Table 6).

**Table 6:** Breakdown of SF-36 scores compared against normative data for 8 adult participants. The scores are combined to give the Physical Component Score (PCS) and Mental Component Score (MCS). Scores are displayed as median (inter-quartile range), apart from Mental Component Score and Physical Component Score, which are displayed as mean (SD). The published norms are displayed as mean (SD)

Domain	Score	UK healthy norms	*p* value
Physical Function	95.00 (13.75)	94.03 (12.36)	1.000
Role – Physical	100.00 (4.69)	93.89 (13.34)	0.187
Pain	92 (23.50)	87.13 (16.56)	1.000
General Health	59.5 (30.25)	78.37 (15.61)	*0.012*
Energy/Vitality	71.88 (37.5)	62.97 (17.21)	0.779
Social Functioning	100.00 (18.75)	88.33 (18.37)	0.660
Role – Mental	100.00 (12.50)	89.64 (16.85)	0.187
Mental Health	75.00 (23.75)	75.29 (16.15)	0.888
Summary			
PCS	53.27 (5.07)	53.64 (5.88)	0.184
MCS	51.23 (11.36)	51.28 (9.01)	0.843

Statistically significant values are indicated in iatalic

## Discussion

This is the first UK study examining a homogenous cohort of congenital agammaglobulinemic patients in order to determine pulmonary health and health-related QoL. Since the first description of congenital agammaglobulinemia by Bruton, such patients have been treated with immunoglobulin replacement and antibiotic prophylaxis [26, 27]. Immunoglobulin replacement has been refined from intramuscular administration, intravenous replacement and now commonly subcutaneous administration [28]. Effective treatment helps prevent significant life-threatening infections, but given the role of B lymphocytes in antigen presentation, and the fact that only IgG is replaced, significant sequelae may continue to develop in these patients, even when receiving immunoglobulin substitution therapy.

This study clearly shows that, despite treatment, patients with congenital agammaglobulinemia are not infection free and retain a higher than average susceptibility to mucosal infections. Although IgG is effective to some degree at preventing mucosal infections, our data suggest that this alone is not satisfactory at controlling mucosal and respiratory infections. This would seem logical given the absence of IgA and IgM in immunoglobulin replacement therapy, which plays the major role in protecting mucosal surfaces. It may also be due to using IgG from a different population donor pool (e.g. the USA) highlighting the different strains of bacteria to which they are exposed. It may also be possible that donor immunoglobulin exhibits less affinity than immunoglobulins produced in local pulmonary lymphoid tissue [29]. Other studies have suggested IgG trough levels should be targeted to prevent breakthrough infections and complications in individual patients rather than trying to achieve a particular trough IgG level [30, 31]. However, in our cohort, of the 6 patients who had evidence of worsening lung disease, 4 had IgG trough levels >10 g/dl, perhaps hinting at the limitations of current treatment strategies even with optimized IgG therapy. Our data suggests that once bronchiectasis has developed, its progression is very difficult to control. It is possible the major determining factor in reducing lung disease is early diagnosis and the prevention of bronchiectasis developing at all in these patients.

Nearly all patients with proven bronchiectasis (6/8) showed progression of their disease despite IgG therapy, one of whom proceeded to lung transplantation. The notes for these patients record only recurrent low-grade infections. Previous reports have shown repeated, albeit low-grade, infections in the sinopulmonary tract of patients with agammaglobulinemia can lead to significant and progressive lung disease, and our results appear to substantiate this [32]. We believe that regular HRCT scans play an important role in the routine follow-up of these patients, not only in the detection of lung disease in patients with apparent low-grade infections but also in the monitoring of the progression of disease and further optimization of IgG therapy.

The majority of patients experience respiratory symptoms to varying degrees (irrespective of whether or not a formal diagnosis of bronchiectasis was made), which has led to a reduced activity level and negative impact on their lives. There is scant previous literature on self-reported HRQoL in patients with XLA and autosomal recessive agammaglobulinemia. Our study clearly shows the majority of patients have significant respiratory symptoms, which negatively impact on their HRQoL. This is supported by work from Hurst et al., who found in a small cohort of patients with primary antibody deficiency the impact of respiratory health is a major contributing factor to the patient’s QoL [21]. The SF-36 data showed a general quality of life comparable with a UK healthy population except for the component ‘General Health’ which was significantly worse in the agammaglobulinemia group. Similar quality of life scores have been found in other studies examining QoL in adults with XLA. This contrasts with QoL in children with agammaglobulinemia which has been reported as significantly worse than the healthy population and other chronic conditions such as diabetes [13, 33].

Like other published datasets, our cohort is small and cared for by a single center for excellence, making statistical analysis difficult and potentially masking any differences in QoL or clinical characteristics. On a larger national scale, other differences in QoL may be found. Ascertaining this understanding is vital since children born today with congenital agammaglobulinemia can be expected to live into adulthood, and therefore, effective treatment should be provided. Other studies have shown that living with XLA directly impacts a patient’s future family planning decisions and potentially that of carrier relatives [6, 13]. For patients to be able to make informed decisions regarding family planning, they must have up-to-date and accurate information about treatment and prognosis. Our study is also limited by an incomplete nature of historical adult records, so ascertainment of historical infection rates is not possible. However, notes and data pertaining to modern management strategies were available, making meaningful comparison possible. For the 2 patients with ‘stable’ bronchiectasis, it was not possible to ascertain whether or not this developed pre- or post-diagnosis of congenital agammaglobulinemia.

Our study shows that despite current treatment regimens, patients who still suffer frequent and recurrent infections are unable to maintain predicted normal lung function, and this therefore means that patients with congenital agammaglobulinemia still have a lower than normal QoL. The aim of treatment for patients with congenital agammaglobulinemias should be to maintain a quality of life equal to that of a healthy population [33]; our pilot study suggests we are some way short of this target. We propose to extend this research to a large, national cohort. These data are vital to help evaluate future treatment strategies such as gene therapy and hematopoietic stem cell transplantation and help evaluate the implantation of a newborn screening program [34–36]. Further work is needed to evaluate and optimize other interventions, specifically prophylactic antibiotics and physiotherapy. Future therapeutic research should be targeted towards tackling the frequent mucosal infections, by either looking at other immunoglobulin isotypes or restoring B lymphocyte function.
